# Clinical and Radiological Outcomes of Corrective Surgery on Adult Spinal Deformity Patients: Comparison of Short and Long Fusion

**DOI:** 10.1155/2019/9492486

**Published:** 2019-03-03

**Authors:** Koichiro Ono, Kazuo Ohmori, Takeshi Hori

**Affiliations:** Center for Spinal Surgery, Nippon Koukan Hospital, Kawasaki 210-0852, Japan

## Abstract

Despite the accumulated knowledge of spinal alignment and clinical outcomes the full corrective surgery cannot be applied to all the deformity patients as it requires considerable surgical burden to the patients. The aim of this study was to investigate the clinical and radiological outcomes of the patients who have received short and long fusion for ASD. A total of 21 patients who received surgical reconstructive spinal fusion procedures and were followed up for at least one year were retrospectively reviewed. Sixteen cases have received spinal corrective surgery that upper instrumented vertebrate (UIV) was thoracic level (group T), or 5 cases were with UIV in lumbar level (group L). Group L had shorter operation time, smaller intraoperative estimated blood loss, and shorter postoperative hospitalization days. Group T tends to improve more in the magnitude of VAS of lumbar pain compared to group L. Improvement of spinal alignment revealed the advantage of long fusion compared to short fusion, in Cobb angle, sagittal vertical axis (SVA), lumbar lordosis (LL), PI-LL C7 plum line (C7PL), and center sacral vertebral line (CSVL). Pelvic tilt (PT) did not differ between the groups. Disc lordosis was the most acquired in XLIF compared to TLIF and PLF and maintained one year. There were 9 adverse events, 3 cases of pulmonary embolism (PE), one case of delirium, and 6 cases of proximal junctional kyphosis. Current study elucidated that long fusion, UIV, is thoracic and can achieve better spinal alignment compared to short fusion, UIV, in lumbar. XLIF demonstrated strong ability to reconstruct the deformity on intervertebral space that is better to apply as much intervertebral space as possible. For the ASD patients with complications, short fusion can be one of the options.

## 1. Introduction

In world's fastest aging society, one of the issues for quality of daily livings (QOL) of aging population in Japan is adult spinal deformity (ASD). ASD are associated with broad range of clinical and radiological findings such as progressive spinal deformity, chronic back pain, and neurological symptoms. Pathology of ASD includes primary degenerative scoliosis (“de novo” form), progressive idiopathic scoliosis in adult life, and scoliosis secondary to vertebral fracture and/or asymmetric arthritic disease [1]. Among these, the number of degenerative and secondary scolioses is increasing in Japanese aging society. Advanced ASD presents loss of function, refractory to nonoperative treatment, and therefore requires the surgical intervention.

Surgical intervention for ASD with posterior-only procedure consists of pedicle screws, osteotomies, and transforaminal interbody fusion [2]. For advanced ASD, posterior-only procedure usually requires high volume osteotomies that carry increased technical demands, longer operation time, and greater blood loss and associated morbidity. Anterior procedure predominantly utilized the disc space to reconstruct spinal alignment that also have large surgical burden as posterior osteotomies [3].

In recent years, minimally invasive surgery (MIS) for spinal fusion has become increasingly popular. The extreme lateral interbody fusion (XLIF) [4] uses the dedicated retractor installed from lateral, abdominal, retroperitoneal, transpsoas approach to lateral portion of the intervertebral disc. XLIF demonstrates strong ability to reconstruct the deformity on intervertebral space [5].

In addition to XLIF, the sacral alar iliac (SAI) screw extends from the second sacral segment to the anterior inferior iliac spine and enabled powerful compression and distraction maneuvers against greater sciatic notch [6].

Lastly, rod rotation [7] and cantilever bending technique [8] enabled correction of scoliosis and enhanced lumbar lordosis.

This recent advance in techniques, instruments, and retractors provides the means to achieve a radiographic correction for ASD to improve clinical outcomes [9]. Despite the accumulated knowledge of spinal alignment and clinical outcomes [9, 10] the full corrective surgery cannot be applied to all the deformity patients as they require considerable surgical burden to the patients. The aim of this study was to investigate the clinical and radiological outcomes of the patients who have received short and long fusion for ASD with a minimum of one year of follow-up.

## 2. Material and Methods

### 2.1. Study Design and Patient Population

**Figure 1:**
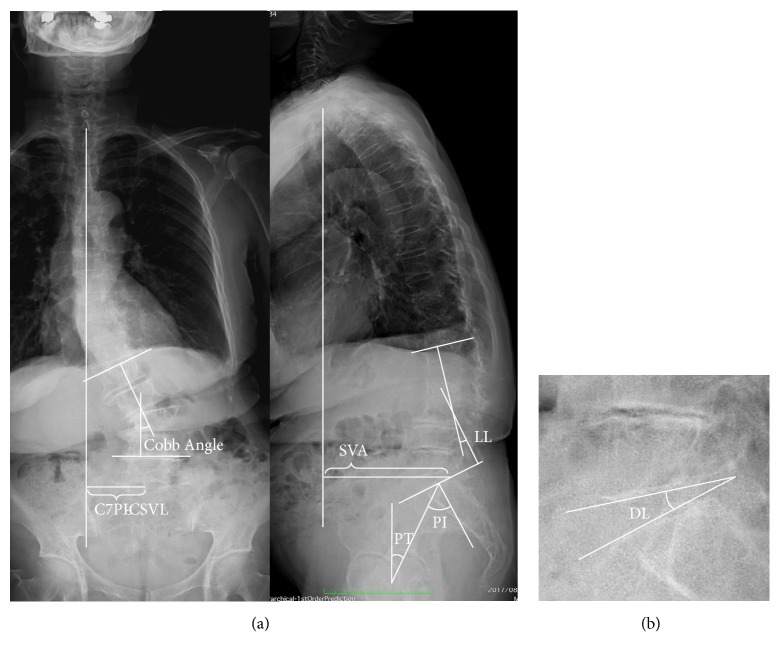
(a) Measurements of sagittal spinal alignment. C7PL-CSVL, C7 plum line to center sacral vertebral line; Cobb angle; SVA, sagittal vertical axis; PT, pelvic tilt; PI, pelvic incidence; LL, lumbar lordosis. (b) DL, disc lordosis.

A total of 21 patients who received surgical reconstructive spinal fusion procedures for their adult spinal deformity at Nippon Koukan Hospital and were followed up for at least one year, from 2014 to 2017, were retrospectively reviewed. Inclusion criteria were symptomatic degenerative adult scoliosis that has failed conservative treatment of patients with Cobb angle of at least 10°, whereas Parkinson disease and deformity due to the vertebral fracture were excluded. The follow-up period was minimum 14.3 months to maximum 57.4 months and average follow-up was 38 months. The mean age was 75.1 years old (65-88 years old), 4 males and 17 females. Of the 21 patients, 16 cases have received spinal corrective surgery that upper instrumented vertebrate (UIV) [10] was thoracic level (group T), or 5 cases were with UIV in lumbar level (group L) (Table 1). Four cases in group T underwent the same day procedure, whereas 12 cases received staged combined anterior and posterior procedures (Table 1). Staged procedures were performed on 7- to 9-day interval; XLIF was performed for the first stage and posterior surgery for the second stage. Clinical and radiographical outcomes were compared between the groups. Also, disc lordosis (Figure 1(b)) was compared among XLIF, transforaminal lumbar interbody fusion (TLIF), or posterior lumbar fusion (PLF). Lastly, introduction of Bendini (Nuvasive, San Diego, CA, USA) was discussed.

### 2.2. Surgical Procedure

In all of staged procedures (12 cases), in the initial surgery, multilevel (2 to 4) XLIF was performed [4]. During XLIF, the retractor attached with the light source was docked on the lateral aspect of intervertebral disc* via* retroperitoneal, transpsoas approach. After the discectomy and trial placement, a lordotic interbody cage (Nuvasive, San Diego, CA, USA) was filled with ReFit (HOYA Technosurgical Corporation, Tokyo, Japan) and autologous bone was harvested from ilium.

For posterior surgery, patients were positioned to prone on the frame and their spine was approached in a standard open fashion. Transforaminal lumbar interbody fusion (TLIF) was performed on intervertebral disc, L1/2 to L5/S1 level, where XLIFs were not performed. After the complete exposure of posterior elements, pedicle screws were placed into all targeted segments. Interspinous ligament resection and facet osteotomies or Ponte osteotomy was performed from L1/2 to L5/S1 among instrumented levels as necessary. Rod rotation [7] and cantilever bending technique [8] were performed to correct scoliosis and enhance lumbar lordosis whenever possible. A curved temporary rod was applied to the convex side of the deformity from T12 to L5 and rod rotation maneuver was performed. A final rod was designed by Bendini spinal rod bending system (Nuvasive, after Mar. 2017) and applied to the concave side with sequential compression and rotation and cantilever technique [8]. Finally, the temporary rod was change to the final rod.

### 2.3. Clinical Assessment

Clinical outcomes were assessed preoperatively and one year and final follow-up using the Japanese Orthopedic Association (JOA) score and the Oswestry Disability Index (ODI). The visual analog scale (VAS) was used for back and leg pain. The medical record was searched for operative data and complications.

### 2.4. Radiological Evaluation

Standing neural anterior-posterior and lateral thoracolumbar films were obtained before surgery, one month and a year after the surgery for assessment. Coronal alignment parameters included Cobb angle and coronal imbalance by plum line deviation (Figure 1(a)). Cobb angle was determined from degree of most superior and most inferior vertebral body (VB) of the scoliotic curve. Coronal imbalance was measured as the distance between a C7 plumb line to the center sacral vertebral line (C7PL-CSVL). Sagittal alignment parameters included lumbar lordosis (LL) from L1 to S1, pelvic incidence (PI), sagittal vertical axis (SVA), and pelvic tilt (PT) (Figure 1(b)). SVA was measured by the horizontal offset from the center of C7 to posterosuperior corner of S1 (Figure 1(b)). Disc lordosis (DL) was determined from degree of inferior line of superior VB and superior line of inferior VB (Figure 1(b)). In addition to comparison study of groups T and L, DL change was compared among XLIF, TLIF, or PLF.

Proximal junctional kyphosis (PJK) was defined as proximal junctional angle and caudal endplate to UIV to the cephalad endplate of 2 proximal vertebrae increases more than 10 degrees. [11].

### 2.5. Data Analysis

For numerical variables, means and standard deviations were calculated, and comparisons were made using a 2-tailed Student's* t*-test. Categorical variables were compared using *χ*^2^ test.

## 3. Results

### 3.1. Clinical and Operative Data

**Figure 2:**
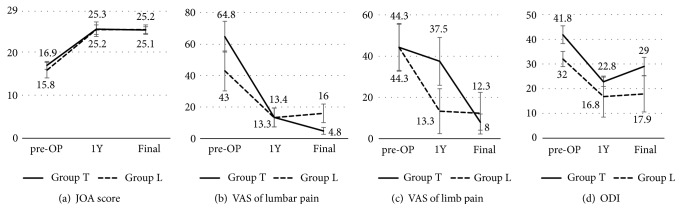
Mean values of clinical outcomes at preoperative, 1 year after the surgery and final follow-up of (a) JOA score, (b) VAS of lumbar pain, (c) VAS of limb pain, and (d) ODI. JOA, Japanese Orthopedic Association; VAS, Visual Analog Scale; ODI, Oswestry Disability Index.

Sixteen patients in group T had a mean age of 76 years (67-88), while 5 cases in group L had 72.2 years (65-77) with no significance (Table 2). Body Mass Index (BMI) (group T versus L = 22.3 versus 23, p = 0.74) and sex (male/female = 2/14 versus 2/3, 0.17) were also similar between groups (Table 2). The mean operation time was significantly shorter in group L (404 versus 285 min., p < 0.01); as a consequence an intraoperative estimated blood loss was smaller in group L (870 versus 137 ml, p < 0.01), and postoperative hospitalization days were shorter in group L (43.9 versus 26.8 days, p = 0.03) (Table 2). There were 9 adverse events recorded. There were three cases of pulmonary embolism (PE) in group T (cases 10, 11, and 14; Table 1). Three cases were all staged cases. One case had episode of delirium in group T (case 17). PJK were developed in four cases of group T (cases 6, 7, 13, and 17; Table 1) and one case of group L (case 2). PJK developed 277.6 days (14-532), on average, after the surgery. PJK occurred 2 weeks after the surgery in the delirium case (case 17). Four of five PJK cases required the additional surgery to extend their instrumentation superiorly. Comparison of improvement ratio in clinical outcomes revealed no significant differences between groups T and L, at one year after the surgery, in JOA score, VAS of leg pain, and ODI (p = 0.49, p = 0.69, and p = 0.7, respectively). However, group T tends to improve more in the magnitude of VAS of lumbar pain compared to group L (p = 0.067). JOA score improved from 16.9 points and 15.8 points (groups T and L), preoperatively on average to 25.3 and 25.2 at 1 year follow-up (p = 0.96) and 25.1 and 25.2 at final (p = 0.96) (Figure 2(a)). VAS of lumbar pain decreased from 64.8 mm and 43 mm preoperatively on average to 13.4 and 13.3 at 1 year follow-up (p = 0.99) and 16 and 4.8 at final (p = 0.13) (Figure 2(b)). VAS of leg pain improved from 44.3 and 44.3 preoperatively on average to 37.5 and 13.3 at 1 year follow-up (p = 0.23) and 8 and 12.3 at final (p = 0.77) (Figure 2(c)). ODI improved from 41.8 and 32 preoperatively on average to 22.8 and 16.8 at 1 year follow-up (p = 0.7) and 25.1 and 25.2 at final (p = 0.41) (Figure 2(d)).

### 3.2. Radiological Data

**Figure 3:**
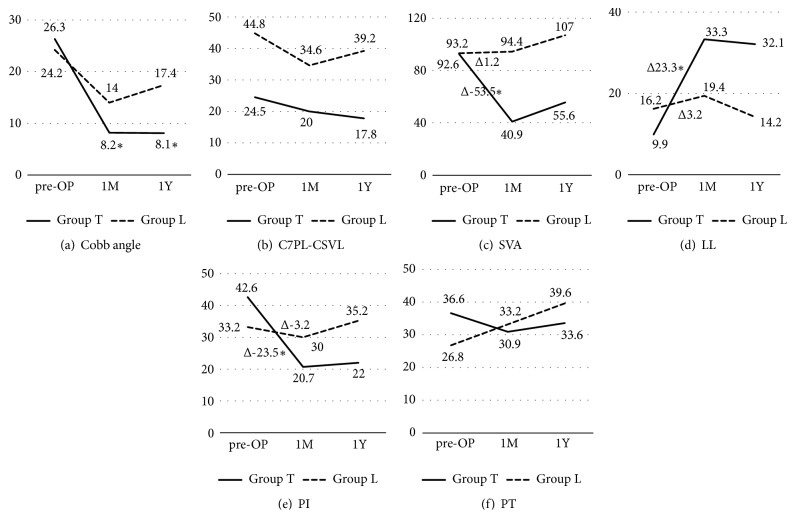
Mean values of radiological measurements at preoperative, 1 month after the surgery and 1 year of (a) Cobb angle, (b) C7PL-CSVL= C7 plum line, central sacral vertical line, (c) SVA = Sagittal vertical axis, (d) LL = lumbar lordosis, and (e) PI = Pelvic incidence. (f) PT = pelvic tilt. *∗* Difference is significant compared to group L.

**Figure 4:**
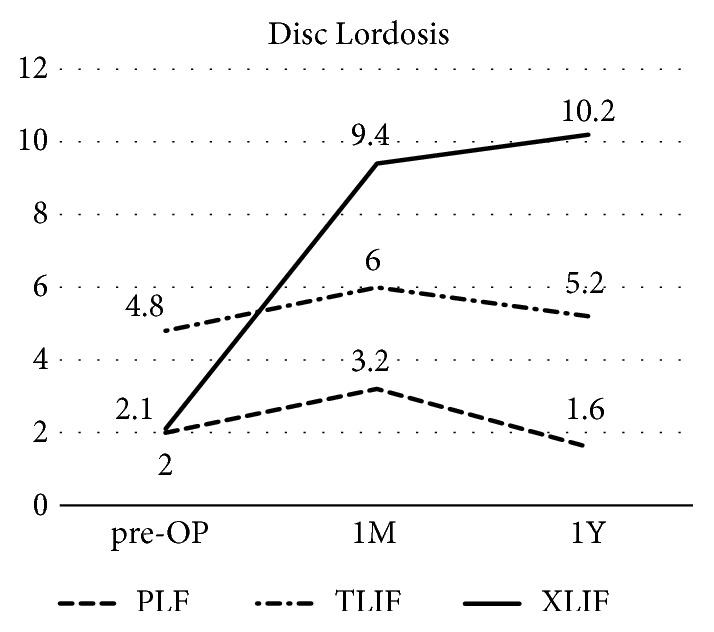
Disc lordosis before operation, 1 month after the surgery, and 1 year.

Improvement of spinal alignment revealed the advantage of long fusion compared to short fusion (Figure 3). On average, Cobb angle improved from 26.3° and 24.2° (group T and L) to 8.2° and 14° at one month after the surgery and 8.1 and 17.4 at one year (Figure 3(a)) (p < 0.01 and p< 0.01, respectively). C7PL-CSVL did not differ between the groups during the course (Figure 3(b)). Global sagittal balance investigated by the SVA changed from 92.6 mm and 93.2 mm to 40.9 mm and 94.4 mm at one month and 55.6 mm and 107 mm at one year (Figure 3(c)) (p < 0.01 and p< 0.01, respectively). SVA improved more in group T, Δ-53.5 mm, compared to group L, and Δ1.2 mm, at one month (p < 0.01). LL changed from 9.9° and 16.2° to 33.3° and 19.4° at one month and 19.5° and 14.2° at one year. LL improved more in group T, Δ23.3°, compared to group L, and Δ3.2°, at one month (Figure 3(d)) (p < 0.01). Similarly, PI-LL changed from 42.6 and 33.2 to 20.7 and 30 at one month, 22 and 35.2 at one year, improved more in group T, Δ-23.5, compared to group L, Δ-3.2°, at one month (Figure 3(e)) (p < 0.01). PT did not differ between the groups during the course (Figure 3(f)). Disc lordosis was the most acquired in XLIF compared to TLIF and PLF (p <0.01) and maintained till one year (Figure 4) (p <0.01).

Four cases in group T and one case in group L developed PJK after the surgery. In group T, patients were divided into PJK and non-PJK group and were compared. Four cases of PJK had larger SVA (82.5 versus 50.2 mm,* p* = 0.11), whereas other parameters did not reach significance. One case in group L had the largest SVA.

## 4. Case

**Figure 5:**
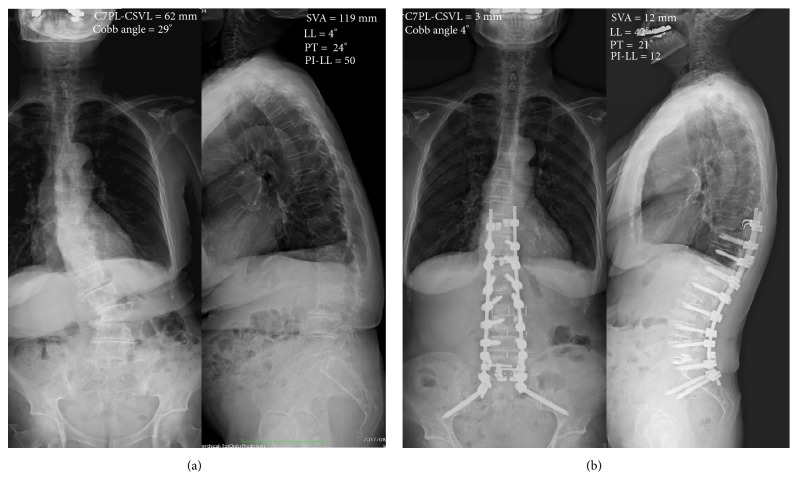
Before and after images of total spine of the case. The after images were 1 month after the surgery.

Eighty-six-year-old female (case no. 21) received staged spinal corrective surgery on her ASD (Figures 5(a) and 5(b)). XLIF was performed on L2/3,3/4,4/5 with 2 hours 29 min., estimated bleeding of 30 ml. Eight days later, open posterior surgery was conducted from T10 to S2 level with 7 hours and 27 min., estimated bleeding of 1100ml. Postoperative hospitalization days were 64 days. Clinical outcomes improved in magnitude of JOA score, VAS of lumbar pain and leg pain, ODI from 23, 64.8, 44.3, and 41.8, preoperatively to 23, 46, and 37, and no data at one month after the surgery, 25, 12, 20, and 17.8 at one year, respectively (Table 3(a)). C7PL-CSVL improved from 62 mm to 3 mm at one month after the surgery and 2 mm at one year. SVA decreased from 119 mm to 12 mm at one month after the surgery and 11 mm at one year (Table 3(b)). Cobb angle improved from 29° to 4° at one month after the surgery and 8° at one year. LL increased from 4° to 42° at one month and 45° at one year. In consequence, PI-LL improved from 50 to 12 at one month and 9 at one year. PT did not change during the course, from 24° to 21° at one month after the surgery and 27° at one year (Table 3(b)).

## 5. Discussion

ASD are associated with broad range of clinical and radiological findings such as progressive spinal deformity, chronic back pain, and neurological symptoms. Previous studies have shown the spinal sagittal alignment and global balance is essential for patients QOL [9, 10, 12, 13]. To achieve proper spinopelvic alignment in the ASD patients, some of operative interventions require more surgical burden for the patient and more technical and physical demand on the spine surgeons. For advanced ASD, posterior-only procedure usually requires high volume osteotomies. Instead of osteotomies, anterior procedure predominantly utilized the disc space to reconstruct spinal alignment that also has large surgical burden [3]. Recently introduced XLIF [4] can be alternative to the anterior procedure. Combined with XLIF and posterior correction and instrumentation, favorable clinical outcomes have been reported [12, 14, 15]. XLIF use the dedicated retractor which requires smaller incision than open anterior procedure and approach from lateral, abdominal, retroperitoneal, transpsoas approach to lateral portion of the intervertebral disc. Surgical field is bright with light source so that retroperitoneal organs and psoas are visible and surgeon can carefully approach the lateral aspect of the disc without bleeding or damaging vital organs. In this manner, XLIF extremely reduced surgical burden compared to the conventional anterior procedure. In addition to reduction of surgical burden, XLIF demonstrates strong ability to reconstruct the deformity on intervertebral space [5]. It restores disc height and indirectly decompression spinal canal. Previous studies have reported XLIF is effective for coronal correction and only mild effect on improvement of sagittal alignment [16, 17]. Therefore, we perform conventional open surgery for posterior procedure to acquire adequate sagittal alignment. Sagittal correction is enhanced using facet osteotomies, rod rotation [7], and cantilever bending technique [8]. Open conventional procedure requires a decent amount of surgical burden that the long fusion needs to consider for their application on the ASD patients with complications. According to our comparison study, group T had longer operation time, intraoperative estimated blood loss, and longer postoperative hospital stay (Table 2). As expected, this result indicated that surgical burden was higher in group T.

It is known that sagittal balance is the most important and reliable radiographic predictor for clinical outcomes [13]. Schwab established the threshold value for the proper spinopelvic alignment in ASD patients, SVA less than 5 cm, PT less than 25°, and PI-LL under 10 [13]. In the current study, improvement of spinal alignments was better in group T compared to group L (Table 3). In group L, the SVA has not changed between before and after; rather it worsened little whereas LL increased after surgery. This might be because the thoracic kyphosis exceeded improved LL. These results indicate that the long fusion is required to achieve adequate sagittal alignment. However, most of the spinal parameters in group T did not satisfy the threshold value of sagittal balance. Correction of coronal deformity is also important in ASD patients. Cobb angle improved in both groups, where group T achieved better correction at one month and one year follow-up. Current study showed correction of Cobb angle was identical to previous report [18]. C7PL-CSVL, however, did not improve to targeted threshold [19]. These insufficient corrections may be because the age of patient of this study was high and bone quality could not tolerate the correction. The other factor for the substandard of the threshold can be the technical issues. For the earlier patients, the rod rotation [7] and cantilever bending technique [8] were not applied, where correction may be inadequate. After these techniques are introduced in combination with Ponte osteotomy and XLIF, postoperative spinal alignment improved compared to previous cases (Figure 5). In addition, disc lordosis was most acquired in the XLIF than TLIF or PLF that XLIFs are performed on many disc spaces as possible (Figure 4). In three cases in group T, Bendini spinal rod bending system was applied for rod bending. This system will save surgeon from the stress of rod bending. Time for rod bending, alignment assessment, and complication such as rod failure needs to be investigated in future.

JOA and some of patients-based outcomes are not significantly different as the spinal parameters. In this study, JOA score is collected by the operators that may cause the bias. Importantly, VAS of lumbar pain improved better in group T compared to group L at the final follow-up, which was parallel to improvement of spinal alignment (Figure 2(b)).

The complication rate was 38%, and there were no mortalities related to the procedure. Seven out of 16 cases developed complications in group T and 1 case out of 5 in group L. Although it was not significantly high, group T seems to have more complications compared to group L. Three cases developed PE in our series (14.3%). The ratio was higher than previous report [18, 20]. After compression stockings and intermittent pneumatic compression device were adopted for precaution during the surgery, no symptomatic PE case was observed. The ratio of PJK was lower in current series (28.3%) [11, 21, 22]. It is known that LL change more than 30° is the risk for PJK [22]. Therefore, lower PJK ratio can be our moderate correction of LL. However, large SVA was found as a risk factor for PJK. These results indicated optimal spinal alignment is required for favorable postoperative clinical outcomes. Further clinical study is required to understand optimal spinal alignment. Additionally, wearing hard corset for more than a year after the surgery might help to avoid PJK.

### 5.1. Study Limitation

This was the retrospective study and the limitation in this study is selection bias and incomplete data. And the number of cases was small to describe conclusion. In future, prospective study with large number of the patients will provide sufficient data to assess defined study.

### 5.2. Conclusion

Current study elucidated that to acquire harmonious spinal alignment and favorable clinical outcomes, long fusion is better than short fusion. XLIF demonstrated strong ability to reconstruct the deformity on intervertebral space that is better to apply as much as possible. For the ASD patients with complications, short fusion can be one of the options [10].

## Figures and Tables

**Table 1:** Case summaries.

Group	Case No.	age	sex	stage	XLIF levels	UIS	instrumented levels	PJK
Group L	1	77	F	1	0	L	L2-SAI	
	2	65	M	1	0	L	L2-SAI	+
	3	67	M	1	2	L	L2-5	
	4	77	F	1	3	L	L2-5	
	5	75	F	1	2	L	L3-5	

Group T	6	78	M	1	0	T	T8-SAI	+
	7	72	F	1	0	T	T10-SAI	+
	8	81	M	1	0	T	T10-SAI	
	9	76	F	1	0	T	T10-SAI	
	10	76	F	2	2	T	T7-SAI	
	11	74	F	2	2	T	T8-SAI	
	12	73	F	2	3	T	T9-SAI	
	13	75	F	2	3	T	T10-SAI	+
	14	67	F	2	2	T	T9-SAI	
	15	74	F	2	3	T	T10-SAI	
	16	72	F	2	4	T	T9-SAI	
	17	69	F	2	2	T	T10-SAI	+
	18	79	F	2	3	T	T10-SAI	
	19	88	F	2	3	T	T10-SAI	
	20	76	F	2	3	T	T10-SAI	
	21	86	F	2	3	T	T10-SAI	

**Table 2:** Clinical and operative variables. BMI = Body Mass Index.

Variable	Group T	Group L	p Value
No. of cases	16	5	
age	76 (67-88)	72.2 (65-77)	0.24
BMI	22.3	23	0.74
Sex (M/F)	2/14	2/3	0.17
Operation time	404*∗∗*	285	<0.01*∗*
Intra-operative estimated blood loss	870	137	<0.01*∗*
Post-operative hospitalization days	43.9	26.8	0.03*∗*

*∗* Statistically significant.

*∗∗* In staged cases, time of second surgery was adopted.

**(a):** 

Clinical scores	pre-OP	1 M	1 Y
JOA score	19	23	25
VAS of lumbar pain	69	46	12
VAS of leg pain	51	37	20
ODI	33.3	-	17.8

**(b):** 

Alignment parameters	pre-OP	1 M	1 Y
C7PL-CSVL	62	3	2
SVA	119	12	11
Cobb angle	29	4	8
LL	4	42	45
PT	24	21	27
PI-LL	50	12	9

## Data Availability

The data used to support the findings of this study are available from the corresponding author upon request.
